# Identifying caregiver-reported modifiable barriers to pediatric oncology clinical trial enrollment and participation

**DOI:** 10.21203/rs.3.rs-7134172/v1

**Published:** 2025-09-15

**Authors:** Rebecca Whitmire, Daniel Wikstrom, Robin Lockridge, Elizabeth Holland, Angelina Van Sprang, Mary Anne Toledo-Tamula, Srivandana Akshintala, Rosandra N. Kaplan, Pamela L. Wolters, Staci Martin

**Affiliations:** Johns Hopkins Sydney Kimmel Comprehensive Cancer Center; National Cancer Institute; National Cancer Institute; National Cancer Institute; National Cancer Institute; National Cancer Institute; National Cancer Institute; National Cancer Institute; National Cancer Institute; National Cancer Institute

**Keywords:** pediatric oncology, clinical trials, health disparities, household material hardship

## Abstract

**Purpose:**

Clinical trial (ClTr) participation is critical to high-quality pediatric oncology care, but significant sociodemographic disparities in trial enrollment exist. Identifying modifiable barriers to participation such as household material hardship (HMH) and limited health literacy (HL), is essential to improving ClTr access. We compared differences in caregiver-reported barriers to pediatric oncology ClTr participation across socioeconomic status (SES) and racial and ethnic groups through a nationwide anonymous online survey of caregivers of children with cancer. We also explored associations between caregiver HL, HMH, and barriers to trial participation.

**Methods:**

English- and/or Spanish-speaking caregivers of children diagnosed with cancer in the last 5 years completed the Research Participation Survey – Caregiver (RPS-C) to assess barriers to ClTr participation, the validated Health Literacy Survey-12 (HLS_19_-Q12) health literacy assessment, and the WellRx questionnaire measuring HMH.

**Results:**

Of 59 participants, 64% were socioeconomically under-resourced, 52.5% identified as racially or ethnically underrepresented, and 62% reported their child had not participated in a ClTr. Under-resourced caregivers reported higher RPS-C barrier scores than adequately resourced caregivers (*z*=3.18, *p*=0.001). There were no significant differences in barrier scores across underrepresented vs represented racial and ethnic groups (*p*=0.203). Lower HL (ρ= 0.557, *p<*0.001) and higher HMH (ρ = 0.562, *p* = 0.006) were associated with higher barrier scores. The most frequently identified barrier was difficulty understanding study risks (>90%).

**Conclusions:**

Under-resourced SES, HMH, and lower HL were associated with increased barriers to ClTr participation. Caregivers reported modifiable barriers that could be targets for intervention to improve ClTr participation and reduce disparities in childhood cancer outcomes.

## 1 Introduction

Despite significant improvements in overall childhood cancer survival rates, sociodemographic disparities in childhood cancer outcomes persist.[1-6] Poverty is associated with disease outcomes in childhood cancers[5-8]. Household material hardship (HMH) is a measure of tangible resource needs that has been operationalized and well-studied as a domain of poverty. [8-11] HMH impacts nearly 30% of children with cancer at diagnosis[12] and increases over a child’s treatment course[13].Clinical trial (ClTr) enrollment provides access to the latest treatment regimens, risk stratification, supportive care, and intensive monitoring; some studies demonstrate clear associations between pediatric oncology ClTr participation and improved patient outcomes, though this not consistent across all diagnoses. [14-16] There are limited data regarding factors contributing to disparities in pediatric ClTr enrollment, especially from the caregivers’ perspective. Furthermore, studies exploring disparities in pediatric oncology ClTr enrollment primarily have focused on racial, ethnic, age, and geographic disparities,[15, 17, 18] which are immutable. Therefore, we completed a cross-sectional anonymous survey among caregivers of children with cancer to identify modifiable barriers to ClTr enrollment and participation. Our co-primary study objectives were to (1) compare caregiver-reported barriers to enrollment and participation on ClTrs between under-resourced SES (socioeconomic status) and adequately resourced SES groups, and (2) compare barriers between underrepresented racial and ethnic groups and represented racial and ethnic groups. Our secondary objective was to compare HMH and HL across under-resourced and adequately resourced groups and across under-represented and represented groups. Finally, our exploratory aims were to (1) determine relationships between HL, HMH, and barriers to trial participation, (2) describe the most frequently reported barriers and facilitators to ClTr enrollment and participation, and (3) examine differences between barriers experienced prior to study and those experienced while on study among caregivers whose child had participated in a ClTr. We hypothesized that caregivers from the socioeconomically under-resourced group and from the racially/ethnically underrepresented group would report increased barriers to ClTr participation for their child compared to their adequately resourced and racially represented counterparts. In addition, we hypothesized that under-resourced and under-represented caregivers would have lower HL and more HMH than adequately resourced and represented caregivers.

## 2 Methods

This study was a cross-sectional, anonymous online composite of surveys including a demographics questionnaire, the HLS19-Q12 HL assessment, the Caregiver Research Participation Survey, and the Well Rx questionnaire screening for HMH (see Measures section for additional survey details). Participant anonymity was prioritized to minimize potential barriers to survey completion. Surveys were accessed through a SurveyMonkey link in English and in Spanish. English or Spanish-speaking adult caregivers of children ages 0–25 years who were diagnosed with cancer within the past 5 years were eligible to participate. Participants also were required to have internet access in order to complete the electronic surveys.

Study recruitment flyers containing the link to the study in text and as a QR code were shared in English and Spanish via websites, social media platforms, and newsletters of advocacy groups, caregiver support groups, local pediatric oncology clinics, and community organizations (Table S1). Flyers also were shared with healthcare professionals throughout the United States, the Children’s Oncology Group (COG) Diversity and Health Disparities Committee, and the Therapeutic Advances in Childhood Leukemia & Lymphoma (TACL) Health Disparities Working Group. The composite survey was active for four months (August 2024-December 2024). With unequal sample sizes with a 2:1 ratio of adequately resourced versus under-resourced groups and represented versus underrepresented groups, recruitment of 39 caregivers would provide 81.8% power to detect an effect size of 1 (mean difference in barriers between groups = 1 SD) with a two-tailed 0.05 significance t-test. We sought to enroll at least 45 caregivers to account for dropouts. To facilitate diverse participant enrollment, we sought to enroll ≥ 40% from an under-resourced group and ≥ 40% from an underrepresented racial or ethnic group.

Once caregivers accessed the survey online, they completed an electronic pre-enrollment eligibility screening and provided informed consent. All measures were administered anonymously; no personally identifiable information was collected. All questions were mandatory to minimize missing data. Respondents who chose not to answer all the questions were not able to proceed with survey completion. All participants were provided with a link to Findhelp throughout the surveys and at the conclusion of the study as a resource to address any identified unmet needs. Findhelp is a vetted national and multilingual database of community support resources by ZIP code addressing social determinants of health (SDOH) including food insecurity, housing instability, access to healthcare, employment and educational support, legal needs, and financial hardship.[19]

This study was deemed exempt from full IRB review (#IRB002015) due to anonymous, non-invasive, and low risk data collection.

### 2.3 Sample groups

Under-resourced SES groups were defined by endorsing household income < 30th percentile on the 2020 US census or by endorsing HMH, specifically food insecurity as measured by the validated Hunger Vital Sign assessment,[20] (2) or by housing instability[21]. Per the NIH 1993 Revitalization Act study, underrepresented racial and ethnic groups were defined as people who identify as African American or Black, American Indian and Alaska Native, Hispanic/Latine, Native Hawaiian, Asian, and other Pacific Islander.[22]

### 2.6 Measures

#### 2.6.1 Sociodemographic Questionnaire

Caregivers answered questions about sociodemographic variables including income, HMH, race, ethnicity, sex, ZIP code, neighborhood characteristics (i.e., urban/suburban/rural), and primary language. Caregivers also provided information about their child’s cancer diagnosis and treatment course.

#### 2.6.2 Research Participation Survey – Caregiver Form (RPS-C)

The RPS-C is a measure created by the research team and designed with collaborative input from patient advocates who previously underwent treatment for childhood cancer. The team consisted of pediatric oncologists, psychologists, a neuropsychologist, and post-baccalaureate research assistants in the National Cancer Institute’s Pediatric Oncology Branch’s multidisciplinary Patient Engagement Committee. The measure first asks caregivers whether their child participated in a ClTr during their cancer treatment. Using branch logic, those who reported past participation were prompted to answer questions about barriers or challenges they considered *prior to enrollment* and barriers experienced *while participating* in the ClTr. If the respondent’s child did not participate in a ClTr during treatment, they noted if they would consider having their child participate in a future ClTr (yes/no). Regardless of their response, they were given the same barrier items as caregivers who had enrolled their child in a ClTr had received about factors that would influence their decision to enroll or not enroll their child in the future. Caregivers of children with past ClTr participation were asked about facilitators that motivated them to enroll their child and benefits the child or family experienced while on study. Questions about barriers and facilitators were answered on a 1–5 Likert scale (1 = not at all true, 5 = very true). The barriers score was calculated by averaging the raw scores across all barrier items in accordance with established methods for analyzing Likert scale data.[23, 24] Respondents additionally were able to list other barriers not described in the survey through an open-ended question. Higher RPS-C barriers scores indicated more barriers to CLTr enrollment and participation. The RPS-C barriers scores demonstrated good internal reliability in our sample of caregivers whose children had enrolled in a ClTr (prior to enrollment α = 0.844, while on study α = 0.885) as well as those who had not previously enrolled in a ClTr (α = 0.818). Facilitator scores also demonstrated strong reliability (α = 0.862).

#### 2.6.3 Health Literacy Survey-12 (HLS_19_-Q12)

The HLS_19_-Q12 is a 12-item validated measure of HL available in English and Spanish that utilizes a 1–5 Likert scale (1 = very easy, 4 = very difficult, 5 = don’t know) to assess participants’ comfort “finding, assessing, and utilizing health services and health information.”[25] For example, respondents are asked how easy or hard it would be to judge the advantages and disadvantages of treatment, follow their doctor’s advice, and make decisions about their health and wellbeing. A total score is obtained by taking the mean of all items and standardizing them to a 0-100 scale. Additionally, scores are categorized as Excellent (> 83.33), Sufficient (66.67 to 83.33), Problematic (50 to < 66.67), or Inadequate (< 50). The HLS_19_-Q12 exhibited strong internal reliability in our sample (α = 0.92).

#### 2.6.4 WellRx Screening Tool

The WellRx is a 13-item screening tool widely utilized in healthcare settings among English and Spanish-speaking individuals to assess for HMH that may impact families across four domains: food security, economic stability, education, and neighborhood/physical environment. The measure employs yes/no items to assess HMH and recent health services utilization.[26] While there is not a validated method of scoring this measure, we chose to sum items 1–8 to calculate a composite score, with higher scores indicating more HMH. Items 9–13 ask about healthcare utilization and unmet needs beyond the scope of HMH and therefore were not included in analyses. This abbreviated WellRx (items 1–8) exhibited strong internal reliability in our sample (α = 0.80).

### 2.8 Data Analysis

Data were cleaned using R[27] and analyzed using SPSS version 29.[28] Descriptive statistics were calculated for all variables. Results of the Shapiro-Wilk test for normality indicated that some variables were not normally distributed (e.g., RPS-C barrier scores among caregivers whose children had not been on a past ClTr, WellRx Sum; *p*s < 0.05). Therefore, nonparametric tests were used in relevant analyses. Because the same barrier items were given to caregivers regardless of whether their child participated in a ClTr, past ClTr participation was examined as a covariate in exploratory analyses. To analyze our primary and secondary objectives, we used Independent Samples t-test or Mann Whitney U tests. To analyze exploratory objectives, we conducted Pearson or Spearman correlations, Independent Samples t-tests or Mann Whitney U tests, ANCOVA or Quade nonparametric ANCOVA, and the Kruskal-Wallis test to assess relationships between sociodemographic characteristics, HL, and RPS-C composite barrier scores. We computed a paired-samples t-test to examine differences in barriers scores reported prior to enrollment versus while on ClTr. Finally, we calculated descriptive statistics of the RPS-C as a composite score as well as by individual items and subscales.

## 3 Results

### 3.1 Caregiver and child demographics

**Figure 1:**
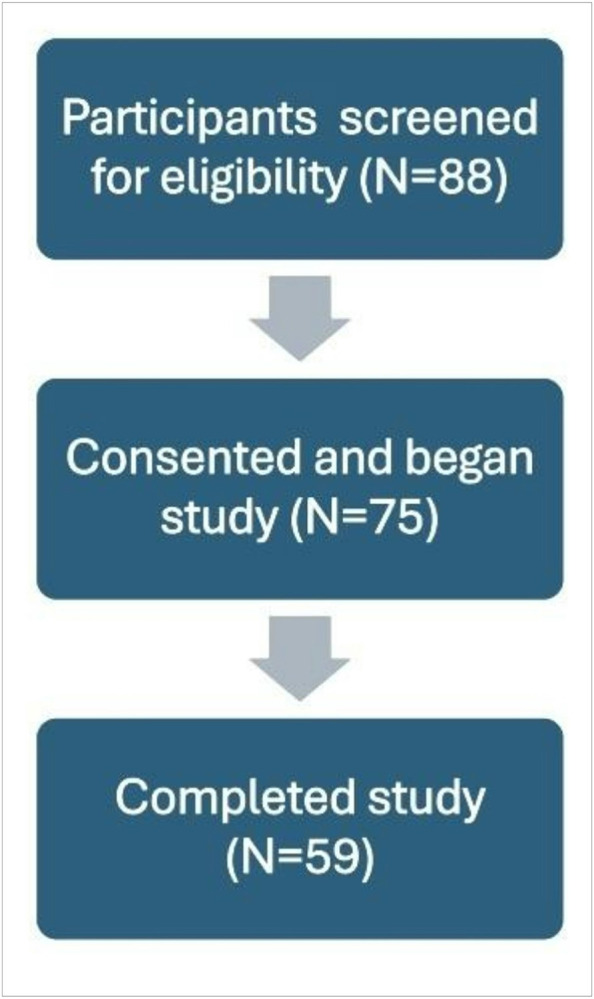
Study consort diagram This figure demonstrates the number of total respondents who requested to complete the study (88), the number of eligible participants who entered the study (75), and the number of participants who successfully completed the entirety of the study (59).

Of the 75 eligible participants who consented to the study and answered at least one survey question, 59 (79%) completed all measures (Fig. 1). The majority (88%) completed the survey in English. Caregivers were mostly female (80%) and the biological parent of their child (98%). The sample represented 15 U.S. states spanning across the country (Figure S1). Over half (64%) of caregivers endorsed HMH and over half (52.5%) of participants identified as an underrepresented race or ethnicity. Most caregivers had private insurance (63%). The median time between survey completion and date of their child’s diagnosis was two years (see Table 1 for detailed demographics).

Children of caregivers participating in the study experienced a broad range of cancers, with leukemia being the most frequently reported diagnosis (35%) (Table 1). Most children were ages 1–10 years (48.3%) or 11–18 years (36.2%) at diagnosis. All children were insured either privately (60%) or publicly (40%) and predominantly lived in two-parent households (69%). Over half (62%) had not participated in a ClTr as part of their cancer treatment.

### 3.2 Descriptive data

Among caregivers whose child had participated in a ClTr, mean RPS-C barriers scores prior to enrollment (*M* = 2.16) were significantly higher than scores reflecting barriers experienced while on ClTr (*M* = 1.75; *t* = 5.95, p < 0.001). The mean HLS19-Q12 score of 66.32 was near the border of Problematic and Sufficient, with 42% of caregivers scoring in the Problematic HL range (Table 2).

### 3.3 RPS-C scores across SES and racial and ethnic groups

Under-resourced caregivers reported higher composite barrier scores (*M* = 2.3) than adequately resourced caregivers (*M* = 1.8, *z* = 3.18, *p* = 0.001). In exploratory analyses, this relationship remained significant while covarying for past ClTr participation (*F* = 5.379, *p* < 0.001). When looking solely across income categories, composite barrier scores were not significantly different while covarying past trial participation (<$39,535 *M* = 2.44 vs ≥$39,535 *M* = 2.11) (*p* > 0.05). No significant differences were observed in composite barrier scores across underrepresented (*M* = 2.11) and represented (*M* = 2.14) racial and ethnic groups when comparing the groups directly (*z* = −0.326, *p* = 0.74) or in exploratory analyses covarying past ClTr participation (*F* = 0.12, *p* = 0.73).

### 3.4 Comparison of WellRx scores across SES and race and ethnicity groups

Under-resourced caregivers (*M* = 2.39) reported significantly higher composite WellRx scores than adequately resourced caregivers (*M* = 0.19, *z* = 3.99, *p* < 0.001). WellRx scores were not significantly different across represented (*M* = 1.29) and underrepresented (*M* = 2.41) racial and ethnic groups (*z* = 1.6, *p* = 0.12).

### 3.5 Relationship between WellRx scores, HL scores, and RPS-C scores

HL scores were negatively correlated with composite RPS-C barrier scores (*r* = 0.506, *p* < 0.001), meaning lower caregiver HL was associated with increased barriers to ClTr enrollment and participation. Results of the Kruskal-Wallis test indicated significant differences in composite RPS-C barrier scores across HL categories (*H* = 13.57, *p* < 0.01). Post-hoc comparisons showed that caregivers with Excellent, Sufficient, and Problematic HL scores had significantly lower barrier scores than those in the Inadequate HL category (all *p*s < 0.01). HL scores did not differ across SES groups or race groups (*p*s > 0.05). In terms of WellRx scores, more HMH were associated with higher composite RPS-C barrier scores (*rho* = 0.562, *p* = 0.006).

### 3.6 Caregiver-reported barriers and facilitators of ClTr participation

For caregivers whose children had participated in a ClTr, the most frequently reported barriers identified before enrollment were concerns about adverse treatment effects (77%), concerns about the treatment being adequately studied (77%), and difficulty understanding the study (73%). Once enrolled, the most frequently reported barriers were about potential adverse effects of treatment (82%) and concerns about the treatment being adequately studied (64%), their child receiving a placebo (55%), difficulty paying for food/rent/transportation/bills at home (55%), and lack of childcare or care for another family member (55%). For caregivers whose child had not enrolled on a ClTr, the vast majority worried about their child taking a treatment that had not been well-studied (92%), experiencing side effects (89%), and receiving a placebo (89%, Figure S2). See figures S2-S5 for distribution of RPS-C barriers and facilitators. Nine caregivers provided free-text responses about barriers to trial participation including lack of an available trial, difficulty understanding study information, transportation requirements, and insurance barriers. These free-text responses mirrored existing RPS-C items.

Regarding facilitators or factors influencing their decision to have their child participate on a trial, caregivers most frequently reported that their child’s oncologist recommended the study (91%) and the opportunity to help medical/research communities learn more about their child’s condition (91%). Four caregivers re-iterated in the free-text responses they were motivated to enroll their child because their child’s oncologist recommended it, there were a lack of other treatment options, and because they believed the treatment would be beneficial and safer for their child. Caregiver-endorsed benefits for their child included receiving treatment (86%) and their child learning more about their cancer (82%). Regarding family benefits, caregivers most frequently reported learning more about their child’s cancer (77%) and meeting other families whose child had a similar diagnosis (64%, Figure S5).

## 4 Discussion

Our findings demonstrate a strong relationship between SES (poverty/HMH) and caregiver-reported barriers to pediatric oncology ClTr enrollment. HMH scores were also positively correlated with barriers to ClTr enrollment independently from household income. Additionally, many caregivers identified specific aspects of HMH, such as difficulty affording food, rent, and bills at home as barriers that made it difficult to consider ClTr enrollment and to continue ClTr participation if they did successfully enroll (Figures S2-S4). Poverty has known associations with adverse outcomes and decreased OS in childhood cancer.[7, 8, 29] Prior studies conducted in healthcare settings have demonstrated that both poverty and HMH are modifiable through linkage to established anti-poverty government measures and community resources,[9-11] and that amelioration of poverty and HMH are associated with improvements in childhood health outcomes.[30-33] Systematic screening of SDOH including HMH and financial strain from diagnosis through survivorship is strongly recommended in pediatric cancer care.[34, 35] Our findings underscore the importance of such screenings and associated interventions. Future research should prospectively assess whether systematically identifying and providing resources to address HMH diminishes known disparities in ClTr participation and whether it improves childhood cancer outcomes.

Our findings additionally elucidate a relationship between caregiver HL and barriers to ClTr enrollment. Importantly, caregivers in the Inadequate HL category had higher barrier scores than each of the other three categories (Problematic, Sufficient, and Excellent), suggesting that even some improvement in HL may help overcome barriers to clinical trials participation and those with the lowest HL level should be prioritized for intervention. HL is a known modifiable risk factor for health disparities[36] that is negatively correlated with important cancer outcomes such as oral chemotherapy adherence in adult patients[37], and associated with ethical ClTr considerations such as informed consent in pediatric oncology populations.[38] HL is often lower among those of lower SES, racial and ethnic minorities, and those who speak languages other than English.[39] While there is evidence that HL interventions can improve pediatric clinical outcomes and healthcare utilization in other populations,[40-41] HL-based interventions within pediatric oncology populations and specifically regarding their impact on ClTr participation are only beginning to emerge.[42] Our study consistently identified caregiver misconceptions about pediatric oncology ClTr methodology as barriers to participation, such as concerns about their child receiving a placebo instead of treatment. As such, one HL intervention to be studied in the future could focus on provider communication and counseling around pediatric oncology ClTrs as a means of improving caregiver HL.

Our study did not identify an association between race and ethnicity and barriers to ClTr participation despite known health disparities among these groups.[4-6] All participants in our underrepresented racial and ethnic group were also socioeconomically under-resourced, which limited our ability to fully delineate the impact of race on caregiver-reported barriers to ClTr participation compared to the impact of poverty/HMH. This pattern is consistent with known complex interactions among race, poverty, and their collective impact on health disparities.[36, 43] Additional studies are required to investigate whether and to what extent SES contributes to known disparities in childhood cancer outcomes by race.

Strengths of this study include that our study sample was socioeconomically, racially, and ethnically reflective of the populations who have been underrepresented to date in pediatric oncology ClTrs [14, 17]. Methodological strengths include a primary focus on the caregiver perspective, a survey completion rate (79%) congruent with expected online survey completion rates[44] as well as strong internal reliability of the RPS-C, WellRx, and HLS_19_-Q12 measures. This study also facilitated household-level assessment of poverty and HMH within the pediatric oncology population, which provides opportunity for future implementation and assessment of targeted anti-poverty interventions to reduce childhood cancer health disparities.

Study limitations include the potential for self-selection bias by recruiting partially through pediatric oncology support groups as these caregivers may have been more open to sharing their experiences than caregivers without support group participation. As mentioned previously, the lack of racially underrepresented caregivers who were adequately resourced highlights a need for broader recruitment in future studies. Additionally, the cross-sectional nature of this study prohibited follow-up to learn how caregiver HMH, HL, and barriers to ClTr participation evolve over their child’s treatment course; future studies could prospectively follow families from diagnosis through clinical trial decision-making and end of participation with interval assessments of HMH, HL, and barriers. Participant anonymity made it difficult to confirm whether clinical trials were available for each of the 41% of participants who said their doctor did not discuss a trial with them, though for all but three of the caregivers who endorsed this barrier, a COG trial was not open for their child’s reported cancer during the year of their diagnosis. Study surveys only were able to be offered in English and Spanish. Provider-patient language discordance is a known barrier to pediatric oncology ClTr participation[17, 45] suggesting that future studies should assess barriers to ClTr participation in other languages to better capture these experiences within a population that does not currently have equitable access to pediatric oncology clinical trials. Finally, caution should be used when interpreting results of the RPS-C and the WellRx, since scoring methods for these measures have not been validated.

In conclusion, studying a socioeconomically, racially, and ethnically diverse sample of caregivers of children with cancer across the country demonstrated that low SES as measured by household income and HMH was significantly associated with increased caregiver-reported barriers to pediatric oncology ClTr enrollment and participation. Low caregiver HL was also associated with increased caregiver-reported barriers to ClTr participation. Importantly, modifiable barriers were identified, including understanding trial risks and benefits, HMH, and other SDOH such as missing school/work, transportation barriers, and lack of access to childcare. Further studies are warranted to explore which barriers may provide the most high-impact opportunities for intervention and to prospectively assess whether systematically identifying and addressing HMH, poverty, and caregiver HL can improve known disparities in ClTr enrollment and in childhood cancer outcomes.

## Supplementary Material

This is a list of supplementary files associated with this preprint. Click to download.

Supplementalinformationforreviewandpublication2.docx

Table1.docx

Table 1 is available in the Supplementary Files section.

## Figures and Tables

**Table 2.** Descriptive statistics for study measures

Study Variable	N	Median	Mean	SD	Range	Skewness	Kurtosis
RPS-C Composite Barriers Score^a^	59	1.98	2.1366	0.59	1.13–4.47	1.186	2.572
RPS-C Barriers subscale: no ClTr experience^b^	37	1.94	2.1256	0.61	1.41–4.47	1.662	4.40
RPS-C Barriers subscale: pre-enrollment barriers^c^	22	2.06	2.1551	0.57	1.13–3.15	0.24	−0.808
RPS-C Barriers subscale: barriers on study^d^	22	1.67	1.7455	0.56	1–3	0.683	−0.037
RPS-C Facilitators subscale: facilitators on study^e^	22	2.5	2.5545	0.76	1.4–4.2	0.305	−0.205
WellRx Score	59	0	1.61	2.068	0–7	1.079	0.049
HLS_19_-Q12 Score	53^f^	66.67	66.32	18.69	19.44–100	−0.305	0.201
Excellent Health Literacy (> 83.33)	9						
Sufficient Health Literacy (66.67 to 83.33)	13						
Problematic Health Literacy (50 to < 66.67)	22						
Inadequate Health Literacy (< 50)	9						

a Scores reflect barriers experienced prior to enrollment among caregivers whose child had been on a ClTr and barriers expressed by caregivers whose did not enroll their child on a ClTr.

b Scores reflect anticipated barriers to enrollment expressed by caregivers whose child had not been on a ClTr.

c Scores reflect barriers experienced prior to enrollment among caregivers whose child had been on a ClTr.

d Scores represent barriers experienced during trial participation.

e Scores represent facilitators and reasons for enrollment among caregivers whose child had been on a ClTr.

f Per HLS_19_-Q12 scoring instructions, scores are invalidated if caregivers provide “I don’t know” responses on > 20% of the items (n = 6/59 caregivers).

## Abbreviations

ALL: Acute lymphoblastic leukemia
AML: Acute myeloid leukemia
COG: Children’s Oncology Group
ClTr: Clinical Trial
EFS: Event-free survival
HL: Health Literacy
HLS_19_-Q12: Health Literacy Survey-12
HMH: Household material hardship
OS: Overall survival
RPS-C: Research Participation Survey- Caregiver
SDOH: Social determinants of health
SES: Socioeconomic status
ZIP: Zone Improvement Plan
